# Hydatid Cysts of Parotid Glands- Diagnosis, Treatment and Recurrences

**DOI:** 10.29337/ijsp.154

**Published:** 2021-07-27

**Authors:** Sabah Abdul Rasool Hammoodi, Kamal Turki Aftan, Mohammed Rhael Ali

**Affiliations:** 1 Department of Oral and Maxillofacial Surgery, College of Dentistry, University of Anbar, Anbar, Iraq; 2 Department of Oral and Maxillofacial Surgery, College of Dentistry, University of Tikrit, Tikrit, Iraq

**Keywords:** hydatid cysts, parotid hydatidosis, parotidectomy

## Abstract

Hydatidosis (Echinococcosis) is a disease caused by infestation of hydatid cysts in any organ of body but mainly liver (70% of cases). Hydatidosis of salivary glands is rare and necessitate computerized tomography for diagnosis while fine needle aspiration remains controversial procedure.

**Materials and methods:**

6 patients diagnosed with hydatid cysts of parotid glands. These cases were admitted and treated at the maxillofacial surgery Clinic of the “AL-Ramadi” Hospital in Iraq. 5 patients were female and 1 male with age group was between 30–50 years. The patients complained of painless unilateral swelling in parotid region and who were diagnosed hydatid cysts using CT. All cases were treated by superficial parotidectomy with cystectomy and preservation of facial nerve.

**Results:**

All hydatid cysts are CE1- type with no recurrences were reported in any of these cases. The postoperative edema was the most common complication. Other complications were not seen.

**Conclusion:**

parotid hydatid cyst should be included in differential diagnosis of persistent parotid swelling especially those with history of hepatic hydatid cysts. Computerized tomography is the gold imaging that aid in diagnosis and classification of hydatid cysts. Most cases are CE1 type and Eosinophilia is a sign of concern in some patients. Surgical treatment remains the “gold standard” in therapy.

**Highlights:**

- Hydatidosis of parotid glands is rare but must be included in differential diagnosis of cystic swelling of salivary glands especially those with history of hepatic hydatid cysts.
- The hydatid cysts are classified according to morphology on imaging into 5 types
- Total serum bilirubin, eosinophilia and leukocytosis are seen
- Superficial parotidectomy with removal of hydatid cysts is the treatment of choice in parotid hydatid cysts

## Inroduction

Echinococcus granulosus (hydatid worm) is a parasitic tapeworm that infects humans and animals. It is transmitted to human by ingestion of contaminated food and water which contain eggs of E. granulosus. The human regards intermediate hosts which contain larval stage (hydatid cyst) while carnivores such as dogs are definitive hosts which contain the adult worms of E. granulosus [1].

Hydatidosis (Echinococcosis) is a disease caused by infestation of hydatid cysts in any organ of body but mainly liver (70% of cases) because it is the first organ encountered after passage of hydatid eggs through small intestine. Less frequently, hydatidosis can affect lung (20%) and others (10%) such as brain, spleen and bone. Regarding symptomatology, hydatid cysts are asymptomatic as the cyst grow and mature slowly so the symptoms depend on the organ involved and stage of cyst development [2].

Hydatidosis of salivary glands is rare and uncommon disease but must be included in differential diagnosis of cystic swelling of salivary glands especially those with history of hepatic hydatid cysts. The published literatures show case reports and mostly involve the parotid gland [3,4,5,6].

The diagnosis of parotid hydatid cyst necessitates imaging ultrasound and preferably computerized tomography (CT). Fine needle aspiration cytology (FNAc) is controversial in suspected cases of parotid hydatid cysts [5,6].

The hydatid cysts are classified according to morphology on imaging into 5 types:

CE1: unilocular simple cyst with double line
CE2: multiseptate cyst
CE3: cyst with detached membranes (CE3a) or contain daughter cysts (CE3b)
CE4: heterogeneous hypoechoic and hyperechoic content, no daughter cysts
CE5: cyst with calcified walls

Although there are several modalities for treatment of hydatid cyst including medical therapy, surgical excision and minimally invasive techniques (such as puncture-aspiration-injection-respiration PAIR), Surgical treatment remains the gold therapy for parotid hydatid cysts. the most serious complication of the parotid hydatid cyst is rapture of cysts and seeding of daughter cysts locally or to other organs.

Few articles were published in literature about parotid hydatid cysts and all were case reports [7,8,9].

The aim of this study is to evaluate the diagnosis, principles of treatment and morbidity of parotid hydatid cysts. The end goal of study is how the surgeon can completely remove the hydatid cyst of parotid and prevent recurrence with minimal morbidity and mortality.

## Materials and methods

Between January 2009 and march 2015, 6 patients diagnosed with hydatid cysts of parotid glands. These cases were admitted and treated at the maxillofacial surgery Clinic of the “AL-Ramadi” Hospital in Iraq.

The following parameters were recorded: age of patient, sex, location primary and secondary cysts, duration and clinical features at admission, Laboratory investigations relevant to liver function and E. granulosus infection, imaging (ultrasound and computerized tomography), the surgical treatment and complications.

There were 6 patients enrolled in the study, 5 were female and 1 male. The age group was between 30–50 years. The patients complained of painless unilateral swelling in parotid region and who were diagnosed hydatid cysts using CT (***Table 1***).

**Table 1:** Clinical data of patients.


PATIENT	GENDER	AGE	TYPE OF CYSTS (PRIMARY OR SECONDARY)	CLINICAL FEATURES	TYPE OF CYST

1#	f	32	Secondary in parotid (primary in liver)	Painless, mobile swelling, intact VII	CE1

2#	f	39	Secondary in parotid and brain (primary in liver)	Painless, mobile swelling, intact VII	CE1

3#	f	55	Secondary in parotid (primary in liver)	Painless, mobile swelling, intact VII	CE2

4#	f	47	Primary in parotid	Painless, mobile swelling, intact VII	CE1

5#	f	44	Secondary in parotid (primary in liver)	Painless, mobile swelling, intact VII	CE1

6#	m	49	Secondary in parotid (primary in liver)	Painless, mobile swelling, intact VII	CE2


Regarding clinical features, the most common feature is painless swelling in parotid region. The swelling grow slowly till become noticeable. Five cases were diagnosed with primary hydatid cysts of liver and secondary parotid hydatid cysts. One case was diagnosed with primary parotid hydatid cyst after exclusion of cysts in other organs of body. In primary parotid cyst, the history of swelling was 5 months while the duration of time of diagnosis of secondary hydatid cyst from first surgical procedure for removal of liver cysts vary from 4 to 11 months.

**Figure 1:**
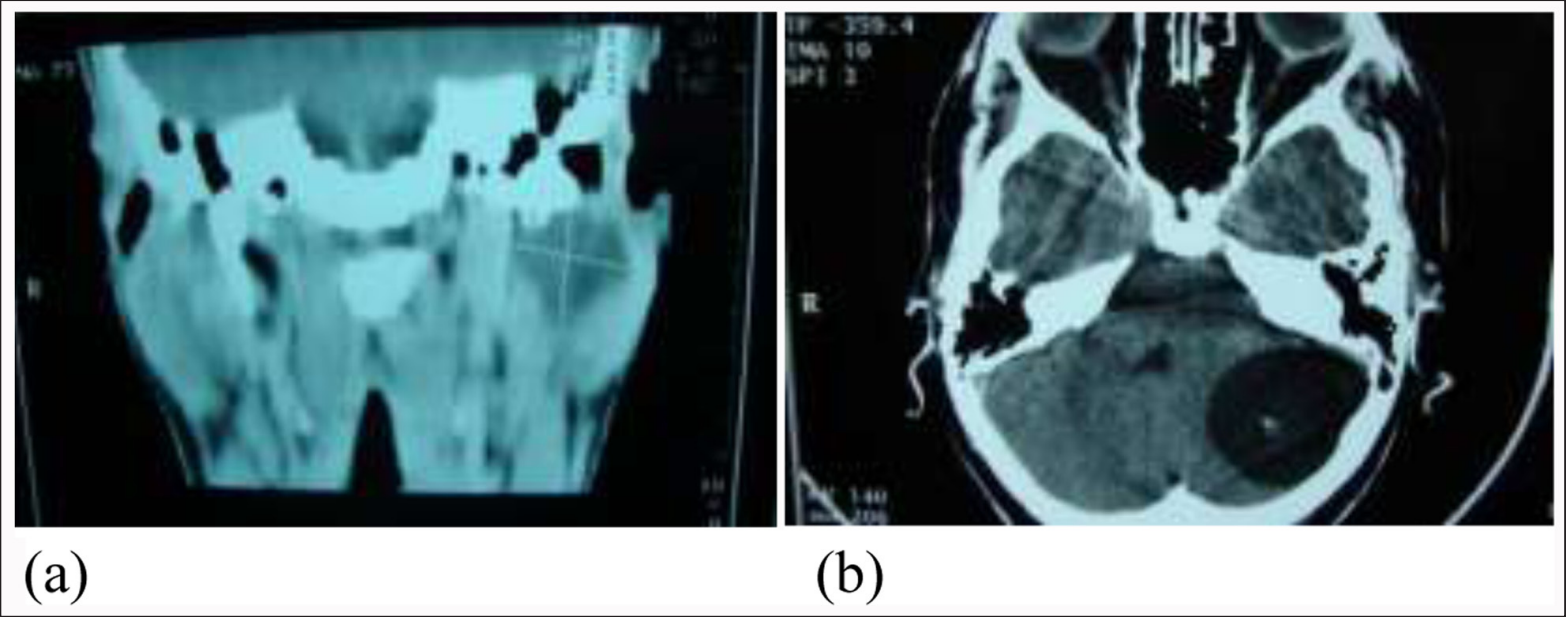
Patient with hydatid cyst pf parotid **(a)** secondary to brain cyst **(b)**.

All cases were investigated mainly by ultrasound and CT which confirm site, size and number of cysts. CT of head, chest and abdomen is recommended. Classification of parotid cysts was performed according to WHO system (***Figure 1***).

Laboratory presurgical investigations including blood count and liver function tests were performed. Immunological investigations (serum IgG anti-Echinococcus granulosus antibody) were not necessary. Fine needle aspiration cytology was not performed in any case due to limited information which can be obtained from crystal fluid which mimic other lesions and risk of anaphylaxis.

**Figure 2:**
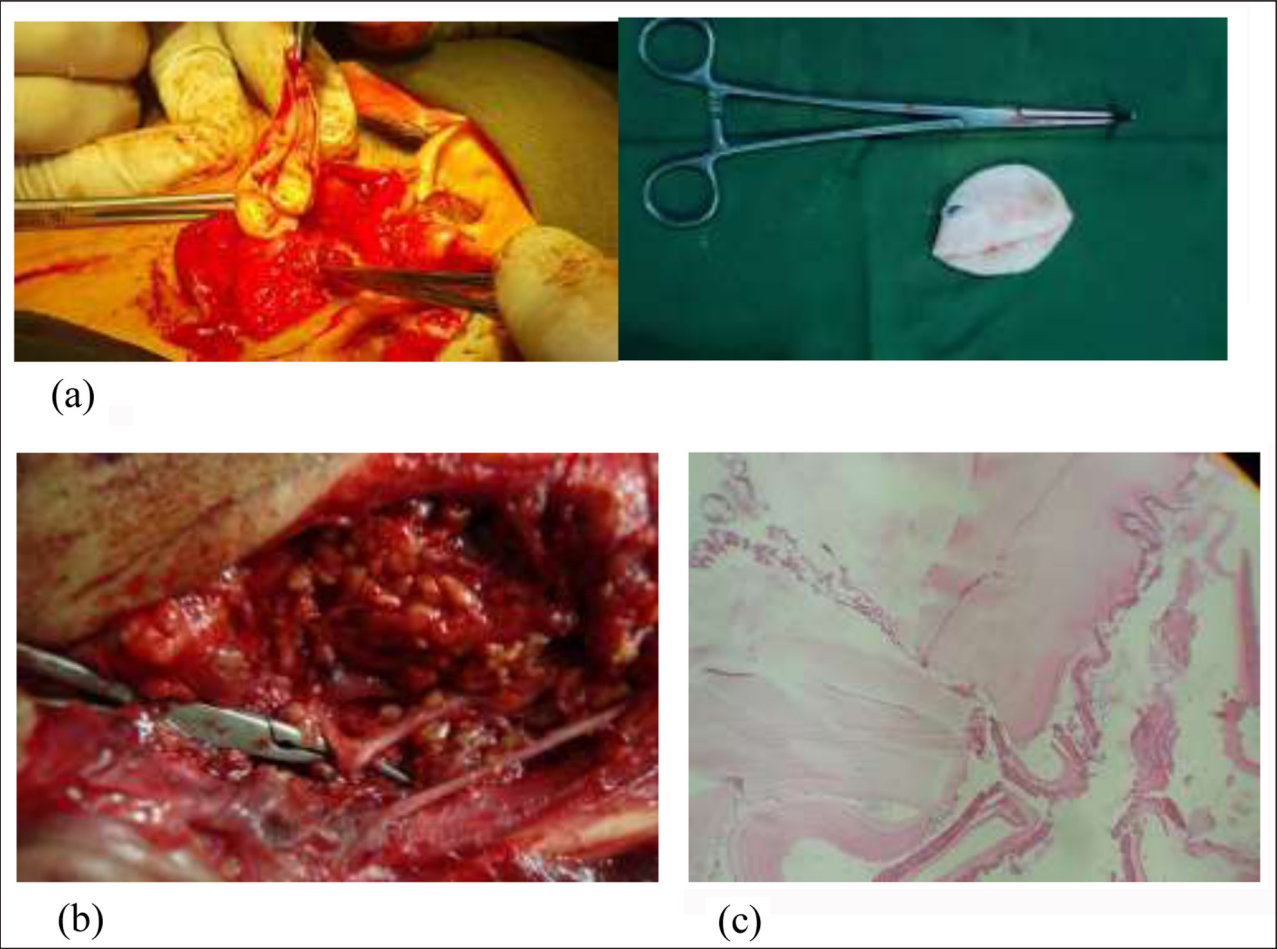
**(a)** Cystectomy. **(b)** Bifurcation of facial nerve trunk. **(c)** Histopathology.

All cases were treated by superficial parotidectomy with cystectomy and preservation of facial nerve. The incision is preauricular (Blair incision) with retromandibular extension. After incision, the superficial musculo-aponeurotic layer is elevated and expose parotid capsule. The capsule is incised carefully. the dissection is carried out between parotid gland and bony external meatus till tragal pointer is identified which point to the trunk of facial nerve. Then the facial nerve is traced anteriorly and dissected from parotid tissue to mobilize and strip the superficial lobe of gland which contains the hydatid cysts. In 3 cases, the cysts were large size and expand the parotid tissue and raptured during dissection. The field was covered previously with cetrimide-soaked pads with continuous suctioning of leaked fluid. 5 patients presented with single cyst while one patient had multiple cysts (***Figure 2***).

Recurrences and any complications like fistula, hemorrhage and facial palsy are recorded in follow up period.

## Results

### Age and gender

A total of 6 patients were included in this study. The age group range was 32–55 years with (SD) was 44.3 year. There were 5 females and 1 male with male to female ratio was 1:5.

### Clinical features

All patients presented with long- standing painless, firm, mobile swelling in parotid region at preauricular area. There was no clinical evidence of compression of vital structures in parotid gland such as facial nerve and retromandibular vein. The skin is intact and is not inflamed. The clinical picture is similar to any cystic lesion. Jaundice was manifested in 2 patients, other systemic features were absent. Five patients had history of liver hydatid cysts and treated surgically and cured while one patient had no previous history. One patient had secondary hydatid cysts in parotid and brain.

### Imaging

**Figure 3:**
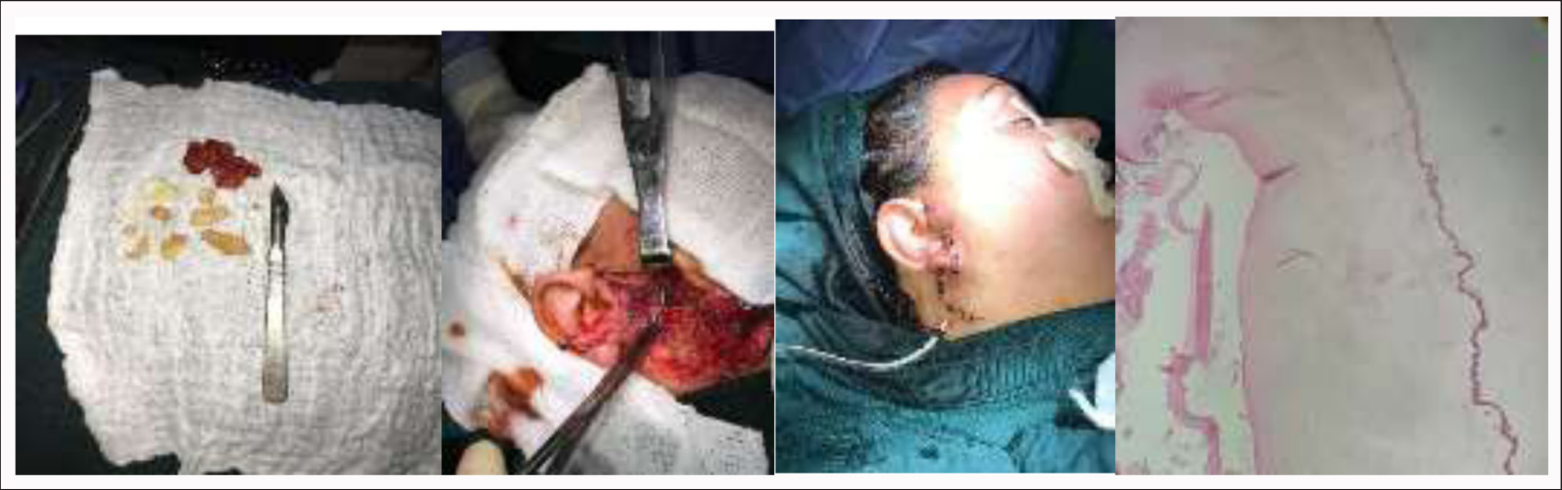
CE2 hydatid cyst of parotid with histopathology.

5 cases of parotid hydatid cysts were CE1 while single case was CE2 (***Figure 3***) according to WHO classification depending on cyst morphology on ultrasound or CT.

Regarding laboratory investigations, eosinophilia was present in two patients, total serum bilirubin level slightly elevated in 4 patients, hepatic enzymes (AST, ALT) and coagulation tests were normal.

All 6 patient were treated surgically that mandate superficial parotidectomy and removal of cysts. Regarding complications, the postoperative edema was the most common complication while facial paralysis was seen in 1 case that was improved with time. Other Serious complications such as fistula, infection, recurrence of disease was not recorded during follow up period.

## Discussion

The incidence of E. granulosus is higher in rural regions where there is contact with farm animals like sheep and horses. So the lifestyle, the level of education and availability of treatment affect the prevalence of disease [10].

Most cases of parotid hydatid cysts were recorded in female and this is probably due to the way of women living in rural cities where there is more contact with infected animals than male such as contaminated fruit and vegetables and consumption of soiled water which contains E. granulosus [8].

Although 90% of E. granulosus cysts occur in liver and lung, 10% of hydatid cysts can occur in any organ of the body. In this study, there were 5 secondary hydatid cysts and one primary hydatid cyst. After ingestion of E. granulosus eggs, the oncospheres pass through small intestine to portal venous circulation and enter the liver. Then the oncospheres (or developing cysts) can pass to the heart and systemic circulation and settle in any organ such as parotid gland. Parotid hydatid cyst may be primary or secondary. The primary hydatid cysts of parotid results from direct settlement of cysts in the gland only without involvement of other organs. however, the secondary cysts of parotid are more common and may result from surgical rapture of primary cysts of liver [9,10].

The frequency with which eosinophilia is encountered IS 25% in the literature versus 39% in the present group. In this study, the average duration of diagnosis of secondary parotid cysts was 7.5 months while in case of the primary parotid cyst, the history of swelling was 5 months. It is known that larvae of hydatid cyst grow slowly about 1–2 cm/year [11]. The hydatid cyst may remain unnoticeable for years till become large enough to be clinically detectable or cause compression of vital structures.

Regarding WHO classification of hydatid cysts whether it is in liver, lung or parotid, most cases (5 patients) show CE1 type cysts which consist of Univesicular fluid collection cyst with double sign seen radiographically. While there was single case consist of multiple daughter cysts (CE2 type). This refers that active stages responsible for most clinical cases which encountered during examination. This comes in accordance with many studies [12,13,14].

Some biochemical parameters may be elevated in cases of parotid hydatid cysts although they were not specific. Total serum bilirubin was elevated in 4 cases due to probable hepatocyte dysfunction from previous liver hydatid cyst. E. granulosus is parasitic infection that may produce eosinophilia and leukocytosis [14].

Superficial parotidectomy with removal of hydatid cysts is the treatment of choice in parotid hydatid cysts. Although it is typical parotidectomy procedure, there are a number of considerations should be taken when suspected hydatid cysts may be encountered. First, avoidance cyst rapture as much as possible by using subcapsular dissection and decompression of cyst if too large. Second, partial superficial parotidectomy may be suffice if small cysts present. All cases, the facial nerve is identified retrograde by identifying tragal pointer.

The complications after superficial parotidectomy in all cases were mild and temporary. No recurrences Hydatid cyst can cause serious complications, such as cyst rupture, with the spread of new cysts and anaphylaxis.

## Conclusion

Parotid hydatid cyst should be included in differential diagnosis of persistent parotid swelling especially those with history of hepatic hydatid cysts. Computerized tomography (CT) is the gold imaging that aid in diagnosis and classification of hydatid cysts. Most cases are CE1 type and eosinophilia is a sign of concern in some patients. Surgical treatment remains the “gold standard” in therapy.
